# The Impact of COVID-19 on Postpartum Depression and the Responsibility of the Healthcare System

**DOI:** 10.7759/cureus.27805

**Published:** 2022-08-09

**Authors:** Sydney DiGregory, Nancy Githere, Kundai Crites, Caroline Rouse, Anthony Shanks

**Affiliations:** 1 Obstetrics and Gynecology, Indiana University School of Medicine, Indianapolis, USA; 2 Obstetrics and Gynecology, Marian University College of Osteopathic Medicine, Indianapolis, USA

**Keywords:** general obstetrics, health disparities and vulnerable populations, mental health services, covid 19, peripartum depression

## Abstract

Peripartum depression is a common complication of pregnancy with the potential for dangerous consequences to maternal and infant health if left untreated. The disorder was previously classified as a global public health issue due to the high prevalence of the disorder and the mismatch between available treatment options and successful completion of those options. The coronavirus disease 2019 (COVID-19) pandemic increased the incidence of mental health disorders globally, with an even greater effect on peripartum mothers. A preliminary study on fetal implications suggests the resulting increased maternal stress and depressive mood symptoms correlates to worsened fetal brain development. The pandemic highlighted existing barriers to the treatment of peripartum mood disorders. The drastic increase in the use of telemedicine as a modality of treatment in response to the public health crisis has the potential to address some of these barriers. Future global disasters are inevitable with peripartum mothers highly susceptible to worsened mental health outcomes. We are thus highlighting the responsibility of clinicians, professional organizations, and policymakers to support, identify, and facilitate the treatment of postpartum depression for this vulnerable population to prevent short-term and long-term repercussions.

## Editorial

Although peripartum depression (PPD) was one of the most prevalent complications before the start of the coronavirus disease 2019 (COVID-19) pandemic, affecting roughly one in seven pregnancies, it remained vastly underdiagnosed and undertreated [1]. PPD is defined as a major or minor depressive episode occurring anytime throughout the pregnancy up to 12 months after delivery [1]. The American College of Obstetricians and Gynecologists (ACOG) has published clinical guidelines for PPD, including the recommendation to administer a validated screening tool, such as the Edinburgh Postnatal Depression Scale (EPDS), at least once in the peripartum period and at the first postpartum visit [1]. Treatment consists of psychotherapy, pharmacologic interventions, or a combination of both [2]. Without treatment, PPD has been associated with negative consequences for the patient and their ability to function as a caregiver [3]. PPD has also been linked with maladapted maternal-infant bonding in addition to poorer outcomes of the child’s social, emotional, language, and cognitive development [3]. Current research suggests that the treatment of PPD improves the negative influence on the bond between mother and infant [4]. While treatment is linked to improved outcomes, barriers to treatment exist, with an increased burden found in non-white patients [5-6]. COVID-19 not only intensified the prevalence of peripartum mood disorders but also the barriers to appropriate access to the healthcare resources needed for treatment. The pandemic prompted a drastic increase in the use of telemedicine for healthcare visits and may have the potential to address some of these barriers [7].

During the COVID-19 pandemic, the mental health of the general population in developed countries was negatively affected, with the prevalence of depression increasing seven-fold, from 3.34% in 2017 to a pooled 25% in 2020 [8]. While this is an astonishing trend, pregnant individuals were even more severely impacted. Prior to the global COVID-19 pandemic, the pooled prevalence of perinatal depression was found to be 11.9%, and postpartum depression was identified among approximately 10-15% of mothers in developed countries [9-10]. As of 2022, the pooled prevalence of depression was subsequently found to be 34% in postpartum individuals, an almost 10% difference from the general population and as high as a 24% increase from the postpartum depression prevalence pre-pandemic [9]. Thus, the COVID-19 pandemic is correlated with a significantly higher prevalence of depression among pregnant and postpartum women, even more so than the general population. This finding indicates that pregnant and postpartum women are a vulnerable population that requires increased attention for mental health concerns during global disasters.

The dangers of PPD on the patient and infant have been well-documented before the pandemic. Negative maternal outcomes involve substance abuse and self-harming behaviors, including suicide. Implications for the infant include poorer outcomes with social, emotional, language, and cognitive development, decreased infant safety practices, as well as maladapted maternal-infant bonding [3]. Importantly, a recent 2022 study found that the consequence of elevated levels of psychological distress during COVID-19 while pregnant correlated to impaired fetal brain growth and development on imaging [11]. The pandemic, thus, had a direct effect on increased mental health disorders with a more dramatic negative correlation on infant development than pre-pandemic. Without increased monitoring and subsequent treatment for pregnant and postpartum mothers, future pandemics and global calamities could have similar negative outcomes for fetal development.

Barriers to initiating treatment include the fear of the stigma that a patient is unable to appropriately function as a mother if diagnosed with a mental health disorder, travel and childcare restrictions, past poor experiences with healthcare professionals, and individuals of low socioeconomic status and racial/ethnic groups [5-6]. Increased social support has historically been a protective factor [5-6]. The nature of pandemics caused high amounts of social isolation and lack of access to in-person routine maternal healthcare, increasing the risk for PPD [10]. As a result, the healthcare community implemented telemedicine into everyday practice. A retrospective study revealed the use of telehealth increased the odds ratio of postpartum visit attendance by 90% and increased PPD screening rates by approximately 20% from pre-pandemic numbers [12]. A rapid review of telehealth services for maternal healthcare concluded that telehealth interventions (e.g., therapy services, anti-depressant dose monitoring) were as effective, and sometimes more than in-person visits for treatment of PPD [7]. Thus, telemedicine strategies have the potential to address certain well-known barriers to care, including limited transportation, a need for childcare services, and managing an appointment during a period of lifestyle adjustment [7,12]. Technology resources can be implemented to increase monitoring of PPD symptoms such as health portals sending automated surveys to patients via email or text message and certified nurse phone calls to check in with higher-risk patients [7]. 

Mental health is a traditionally underfunded and under-resourced field of medicine. Increased attention to mood symptoms is required for pregnant patients at even the best of times but particularly during global crises such as the COVID-19 pandemic. The responsibility for PPD identification and treatment falls on the shoulders of all healthcare system levels. Individual clinicians and healthcare systems must integrate administering a validated screening tool (e.g., EPDS) into the clinic workflow to increase the identification and monitoring of the disorder; strategies include providing the tool for completion in the waiting room or during nurse intake. Telehealth is a potential resource to counteract certain barriers to care as well as minimize postpartum visit no-show rates and increase the opportunity to diagnose PPD. Healthcare systems can track PPD screening compliance and integrate reward programs for clinical teams with high rates of screening tool administration. Healthcare clinicians must have awareness of available resources offered by professional organizations. ACOG currently provides a Postpartum Toolkit containing information for the identification, education, and treatment of PPD [13]. Additional recommendations on best practices to successfully implement telemedicine services may be a topic that professional organizations choose to update. Healthcare funding must be funneled into the now over-burdened mental health system to combat the consequences of the pandemic. Federal and state healthcare policies should implement increased coverage (i.e., Medicaid) for mental health services and prescriptions to treat PPD in the higher-risk, low socioeconomic patient populations. Public health services must focus on initiatives to increase education about PPD for patients to have the ability to recognize alarm signs and symptoms. 

Postpartum depression is easily identifiable and treatable. If the pregnant population is neglected, the dire health consequences for both mother and infant are proportionally affected and cannot be ignored. Global crises similar to the COVID-19 pandemic are likely to occur again in the future. The healthcare system should be collectively prepared to take appropriate action for the increased prevalence of PPD now and in the future.
